# Patient classification in episodic migraine based on data-driven pain curve dynamics

**DOI:** 10.3389/fneur.2026.1853701

**Published:** 2026-10-13

**Authors:** Ana Beatriz Gago-Veiga, Jose L. Ayala, Javier Gálvez-Goicuría, Alicia Gonzalez-Martinez, Sonia Quintas, Alba Vieira, Ancor Sanz-García, Monica Sobrado, Jose Vivancos, Josué Pagán

**Affiliations:** 1 Neurology Department, Hospital Universitario de La Princesa & Instituto de Investigación Sanitaria Princesa (IIS-Princesa), Madrid, Spain; 2 Departamento de Medicina, Universidad Autónoma de Madrid, Madrid, Spain; 3 Computer Architecture and Automation Department, Universidad Complutense de Madrid, Madrid, Spain; 4 CCS: Center for Computational Simulation, Universidad Politécnica de Madrid, Madrid, Spain; 5 Brainguard SL, Pozuelo de Alarcón, Spain; 6 Electronic Engineering Department, Universidad Politécnica de Madrid, Madrid, Spain; 7 Unidad de Análisis de datos, Instituto de Investigación Sanitaria (IIS-Princesa), Hospital Universitario de la Princesa, Madrid, Spain

**Keywords:** cluster analysis, episodic migraine, machine learning, pain dynamics, patient classification

## Abstract

**Purpose:**

Migraine is characterized by heterogeneous manifestations, with pain being its most disabling feature. Although the International Classification of Headache Disorders, 3rd edition (ICHD-3), defines diagnostic criteria for migraine, it does not incorporate temporal dynamics of pain. While studies have described attack-level pain trajectory patterns, the translation of these dynamic features into patient-level stratification remains unexplored. This study aimed to classify individuals with episodic migraine according to pain trajectories and to characterise the clinical features of the resulting groups.

**Patients and methods:**

Patients fulfilling ICHD-3 criteria for episodic migraine participated in a prospective real-time study using a smartphone application to record migraine attacks. Pain onset, duration, intensity, and associated premonitory, concomitant, and postdromic symptoms were documented. A total of 344 migraine attacks from 51 patients were analyzed. Attack-level pain trajectories identified in earlier work were used to construct patient-level feature vectors representing the proportional distribution of attack types. These vectors were clustered using a K-means algorithm to identify patient subgroups.

**Results:**

Fifty-one patients (mean age: 38.8 ± 10.9 years; 90.2% women) recorded 344 migraine attacks. Five patient profiles were identified from the combination of four previously established pain trajectories: high-intensity, mixed pattern, acute-onset, prolonged-intense, and low-intensity. The mixed profile was the most frequent (39.2%), while the remaining profiles showed a clearer predominance of a specific attack type. Most conventional demographic and clinical variables, including age, sex, baseline diagnosis, and monthly migraine frequency, did not differ across profiles. Concomitant symptom burden differed across profiles, with significant differences in nausea, photophobia, and osmophobia after accounting for within-patient clustering and multiple comparisons. The low-intensity profile showed a lower premonitory symptom burden than the high-intensity, mixed, and prolonged-intense profiles, whereas the prolonged-intense profile showed a greater postdromic symptom burden than the low-intensity profile.

**Conclusion:**

Our findings suggest that the way migraine pain evolves over time may help identify clinically distinct patient profiles. Considering pain trajectory adds a dynamic dimension to migraine phenotyping that is not captured by traditional diagnostic criteria and may contribute to improved phenotyping and hypothesis generation for personalized therapeutic strategies. Further studies in larger cohorts are needed to clarify its potential clinical relevance.

## 1 Introduction

Migraine is a common and disabling neurological disorder affecting approximately 14–15% of the global population and representing one of the leading causes of disability worldwide (1, 2). The International Classification of Headache Disorders, 3rd edition (ICHD-3), categorizes migraine into distinct clinical subtypes according to features such as headache frequency and the presence or absence of aura, providing an essential framework for diagnosis and clinical management (1, 3, 4). However, while current classification systems capture key clinical characteristics of migraine, they do not fully reflect the dynamic evolution of pain during individual attacks. Increasing attention has therefore been directed toward a deeper understanding of the temporal characteristics of the migraine pain phase and their potential contribution to migraine heterogeneity.

Although substantial progress has been made in characterizing the variability of migraine attacks, classification approaches incorporating the temporal dynamics of pain evolution remain insufficiently explored in migraine research (5). Recent technological advances have enabled real-time recording of migraine attacks through smartphone applications designed to capture dynamic pain trajectories (6–8). These applications accurately register pain onset, duration, and intensity, as well as associated symptoms, providing a robust framework for data-driven analysis of migraine patterns.

Building on this technological approach, our group previously identified four distinct migraine pain curve patterns at the attack level using real-time smartphone-based data collection (9). These patterns reflected different dynamic profiles of pain evolution during migraine episodes. Subsequently, we demonstrated that these four pain curve types were significantly associated with specific clinical characteristics in episodic migraine patients, supporting their clinical relevance and reinforcing the hypothesis that migraine heterogeneity may be partly explained by differences in pain trajectory patterns (10).

Despite establishing the existence and clinical significance of these four distinct attack-level pain patterns, it remains unclear whether patients themselves can be stratified into meaningful subgroups according to the predominant pain curve pattern of their migraine attacks. In this context, the present study aimed to evaluate whether episodic migraine patients can be classified into distinct subgroups based on the previously identified pain curve patterns. By analyzing real-time data collected through a smartphone application, we sought to determine whether previously identified attack-level pain trajectories can be leveraged to generate clinically meaningful patient-level phenotypes. In addition, we explored the clinical characteristics associated with the resulting patient profiles, including premonitory, concomitant, and postdromic symptoms to further characterize the clinical profiles underlying this stratification.

## 2 Methods

In this prospective study, we aimed to determine whether the pain curve patterns previously identified at the attack level (9) allow meaningful stratification of episodic migraine patients at the patient level, and to characterise the resulting patient groups.

### 2.1 Study design and participants

Patients were consecutively recruited at the Headache Unit of Hospital Universitario de La Princesa over a two-year period based on a confirmed diagnosis of episodic migraine according to the International Classification of Headache Disorders, 3rd edition (ICHD-3) criteria (1). A comprehensive baseline clinical evaluation was performed to confirm eligibility and ensure strict adherence to diagnostic criteria.

Demographic and clinical variables collected at baseline included age, sex, age at migraine onset, cardiovascular risk factors, smoking status, dyslipidemia, family history of migraine, baseline diagnosis, preventive treatment use, body mass index (BMI), and Headache Impact Test-6 (HIT-6) score. Migraine with aura was defined according to ICHD-3 diagnostic criteria. Patients were classified at the patient level as having migraine with aura if they fulfilled formal diagnostic criteria for migraine with aura at any point in their clinical history; otherwise, they were classified as migraine without aura.

### 2.2 Cognitive reserve assessment

Cognitive reserve was assessed using the Cognitive Reserve Questionnaire (CRC), a validated instrument that evaluates educational attainment, occupational complexity, and engagement in cognitively stimulating activities. Total scores were calculated according to the original scoring system. Based on these scores, cognitive reserve was categorized into four levels: 0 = low (<7), 1 = medium–low (7–9), 2 = medium–high (10–14), and 3 = high (>14), reflecting increasing levels of cognitive reserve. For comparative analyses, a dichotomous variable was created (<3 vs. 3), where the 3 category represents individuals with the highest level of cognitive reserve (corresponding to scores >14), while the <3 category includes all lower levels. This operational approach was used to facilitate the identification of clinically relevant differences between patient subgroups.

### 2.3 Prospective attack recording

Following enrollment, participants were included in a prospective observational study and followed for approximately 4–6 weeks. During this period, all migraine attacks were recorded in real time using a dedicated smartphone application specifically designed to capture pain dynamics (9).

The application also recorded concomitant symptoms and acute treatment use for each migraine attack, including the type of rescue medication used and the timing of the first acute medication relative to pain evolution. Pain intensity was recorded using an 11-point numerical rating scale (NRS 0–10) integrated into the application interface. All entries were time-stamped, ensuring prospective and ecologically valid data capture throughout the follow-up period. The resulting dataset comprised detailed longitudinal attack-level records for each participant. For each recorded attack, the presence or absence of aura was also documented, enabling the analysis of aura at the attack level in addition to patient-level classification.

The study population and the data-cleaning procedures followed the cluster-then-classify methodology previously described by our group (9). In that work, the original attack-level records were progressively filtered by removing false events, records unsuitable for pain-curve reconstruction, and patients who did not meet the minimum recording requirements. The present analysis used the resulting patient-level dataset after the corresponding preprocessing procedures, comprising 51 patients and 344 migraine attacks.

### 2.4 Secondary analysis of premonitory, concomitant, and postdromic symptoms

In addition to pain dynamics, the smartphone application enabled recording of premonitory, concomitant, and postdromic symptoms for each migraine episode using predefined symptom lists, with the option to add free-text entries under “other” when applicable. Premonitory symptoms were defined as those occurring before the onset of headache pain, concomitant symptoms as those occurring during the headache phase, and postdromic symptoms as those occurring after resolution of the pain phase. Symptoms could be entered either during the course of the attack or upon completion of the episode.

### 2.5 Identification of patient cohorts based on pain evolution profiles

Patient cohorts were established based on the distribution of the four pain evolution curves previously identified (9). Each patient was modeled as a four-dimensional vector, where each dimension represented the frequency of a specific migraine type divided by that patient’s total number of recorded episodes; thus, the four features summed to 1 for each patient. This feature matrix was processed using an unsupervised K-means clustering algorithm, implemented via the scikit-learn library (version 0.24.2) with K-means++ initialization and 30 random starts. The Euclidean distance was used because it is interpretable. Because Euclidean distance does not generally represent the Aitchison geometry of compositional data, a centered log-ratio (CLR) sensitivity analysis was also performed as a complementary assessment of the potential influence of the distance geometry. We note that direct CLR transformation is not defined when zero proportions are present without an adjustment; therefore, the CLR analysis was used only as a sensitivity check rather than as the primary clustering model.

K-means was selected because the analysis involved a low-dimensional patient-level feature matrix and the method provides an interpretable representation of cohort centroids. Hierarchical clustering and Gaussian mixture models were considered as alternative approaches. Their results were examined in a separate sensitivity analysis; these methods showed partial agreement with the K-means partition, supporting the exploratory nature of the classification while indicating that cluster assignment may depend on the selected algorithm.

Only patients with at least three recorded migraine attacks during the follow-up period were included in the clustering analysis. This criterion was intended to ensure that each patient-level feature vector was based on repeated observations. However, it may have excluded patients with fewer attacks or lower recording adherence and may therefore introduce selection bias.

The unit of analysis for patient-level clustering was the patient, not the individual migraine attack. Thus, the 344 recorded attacks were aggregated within each patient to construct a single four-dimensional feature vector for each of the 51 patients. To assess clustering reproducibility, a patient-level bootstrap resampling was performed 100 times. In each repetition, patients were resampled with replacement, K-means was refitted, and the resulting assignments were compared with the reference solution using the Adjusted Rand Index (ARI).

To evaluate the number of patient groups, K-means solutions with k = 2–10 clusters were compared using WCSS (Within-Cluster Sum of Squares), Silhouette score, Calinski-Harabasz index, and Davies-Bouldin index. Empirical permutation tests with 100 feature permutations were additionally performed to assess whether the observed clustering structure differed from that expected by chance. Because the geometric elbow depends on the evaluated range, sensitivity was assessed by repeating the WCSS elbow calculation.

As individuals experience new migraine attacks, their feature vectors should be continuously updated, and the algorithm reassigns them to the cohort with the closest centroid. This approach ensures an iterative refinement of the patient categorization over time.

### 2.6 Statistical analysis of sociodemographic and clinical variables

Statistical associations between the identified cohorts and patient sociodemographic profiles were evaluated using comparative tests, with *p*-values adjusted for multiple comparisons via False Discovery Rate (FDR) correction. All comparisons were performed at the patient level using the five predefined patient cohorts. Because the cohort sizes were unequal and several cross-tabulations contained sparse or zero cells, selected continuous variables were categorized using the cut-points specified in Table 3 to obtain interpretable contingency tables and avoid reliance on asymptotic approximations that would be unreliable in this sample. Age at migraine onset was categorized as <10 versus 10–39 years using an exploratory analytical cut-point selected to reduce sparse cells in the patient-level contingency table. This categorization was not used to define the patient cohorts and should not be interpreted as a validated clinical threshold. Categorization is an analytical strategy for handling sparse data and does not increase the underlying statistical information; it may reduce statistical power and obscure within-category variation. Where expected cell counts were small, exact tests were preferred.

Homogeneity of variance across groups was assessed using Levene’s test. Normality was evaluated separately using the Shapiro–Wilk test and inspection of Q-Q plots. Where the assumptions of parametric testing were not met, or where group sizes were too small for a reliable assessment, non-parametric alternatives were used. A *p*-value <0.05 was considered statistically significant after the prespecified multiplicity correction.

The analysis incorporated a comprehensive set of variables, including attack frequency, educational level, cognitive reserve, smoking status, age of onset, clinical diagnosis, sex, family history, dyslipidemia, age, and cardiovascular risk factors. Selected variables, including cognitive reserve, smoking habits, age of onset, and diagnosis, were recategorized using predefined or clinically interpretable cut-points to reduce sparsity in patient-level contingency tables. These transformations involved information loss and may have reduced statistical power or obscured dose response and within category relationships. Any regrouping of patient cohorts was treated as exploratory and its results were interpreted cautiously rather than as confirmatory evidence.

Attack-level analyses were conducted separately from patient-level analyses. Because patients contributed multiple migraine attacks, the 344 attack-level observations were nested within 51 patients and were not statistically independent. Attack-level frequencies are therefore reported descriptively as n (%) by pain curve category, with Fisher’s exact *p*-values interpreted as descriptive comparisons.

To account for within-patient dependence, attack-level outcomes were additionally analyzed using logistic Generalized Estimating Equations (GEE), with the relevant categorical grouping as a predictor, patient identifier as the clustering variable, and global differences assessed using Wald tests. When sparse or empty cells prevented reliable GEE estimation, only the descriptive Fisher *p*-value was reported. Logistic mixed-effects models with a patient-level random intercept were used as a sensitivity analysis. Counts of premonitory, postdromic, and concomitant symptoms per attack were analyzed using Poisson GEE models with an exchangeable working correlation structure and patient identifier as the clustering variable. One-way ANOVA was retained as an unclustered descriptive comparison, whereas patient-clustered Poisson GEE provided the dependence-aware global inference for these count outcomes. Individual binary symptoms were analyzed using logistic GEE.

Because acute treatment may modify pain intensity and attack duration, an exploratory sensitivity analysis was performed to assess whether rescue-medication use and timing differed across the five patient profiles. Medication class, use of any rescue medication, number of acute medications per attack, and timing of the first medication relative to the pain peak were evaluated at the attack level while accounting for repeated attacks within patients using GEE models. Binary outcomes were analyzed using logistic GEE, medication counts using Poisson GEE, and continuous treatment timing using Gaussian GEE. Pairwise comparisons were adjusted using the Benjamini-Hochberg FDR procedure.

All analyses were conducted using Python (rpy2 library in version 3.5.17 for Fisher’s exact test, and Pingouin library version 0.5.5 for other patient-level comparisons, and statsmodels version 0.14.6 for the GEE and mixed-effects models). Data were analyzed on a complete-case basis, and no imputation methods were applied due to the low proportion of missing data.

### 2.7 Ethics approval and consent to participate

The study was approved by Hospital de la Princesa’s Ethics Committee (register number: PI-879). All patients signed informed consent forms prior to inclusion in the study. Patient data were anonymized for data analysis. This study was conducted in accordance with the principles of the Declaration of Helsinki.

## 3 Results

### 3.1 Baseline characteristics of the study population

A total of 51 patients were included in the analysis, contributing 344 recorded migraine attacks. Figure 1 shows the workflow illustrating the progression from attack-level trajectories to patient-level aggregation, feature-matrix construction, K-means clustering, validation, and final patient classification.

**Figure 1:**
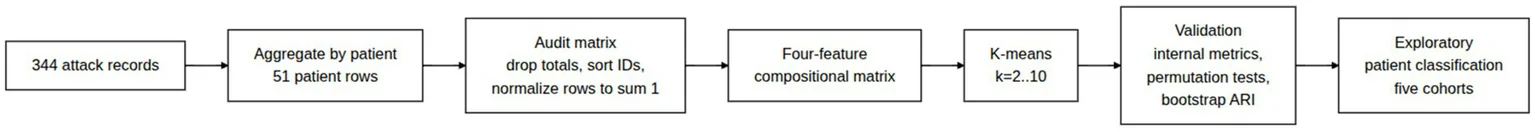
Workflow from attack trajectories to patient classification.

The mean age of the cohort was 38.8 ± 10.9 years, and 90.2% were women. The mean age at migraine onset was 14.36 ± 6.16 years. A family history of migraine was reported in 70.6% of participants. The median Headache Impact Test (HIT-6) score was 63 (IQR (interquartile range) 57.5–65.5), reflecting a substantial impact of migraine on daily functioning. Cardiovascular risk factors were infrequent: hypertension was present in 5.9%, diabetes mellitus in 2%, and dyslipidemia in 11.8% of patients. The median body mass index (BMI) was 23.67 kg/m^2^ (IQR 20.72–27.84). Baseline demographic and clinical characteristics are summarized in Table 1.

**Table 1:** Baseline demographic and clinical characteristics of the study population.

Characteristic	Total sample (*n* = 51)
Age (years) average ± SD	38.8 ± 10.9
Sex [woman *n* (%)]	46 (90.2%)
Migraine onset (years) mean ± SD	14.4 ± 6.2
Family history of migraine (%)	36 (70.6%)
Cognitive reserve: average ± SD	16.1 ± 3.3
HIT-6 median (interquartile range)	63 (57.5–65.5)
Hypertension, %	3 (5.9%)
Diabetes mellitus, %	1 (2.0%)
Dyslipidemia, %	6 (11.8%)
Smoking, %	8 (15.7%)
BMI median (interquartile range)	23.7 (20.7–27.8)

HIT-6, headache impact test; BMI, body mass index.

### 3.2 Pain curve classification

Four distinct attack-level pain curve patterns were identified based on previously characterized pain dynamics (9). These patterns represent different trajectories of pain evolution during migraine episodes and include: (1) high-intensity curves, characterized by consistently elevated pain levels; (2) acute-onset and intense curves, marked by a rapid escalation of pain intensity; (3) prolonged and intense curves, defined by sustained high pain levels over an extended duration; and (4) low-intensity curves, characterized by overall lower pain intensity throughout the attack. These four dynamic patterns form the basis of the subsequent patient-level classification and are illustrated in Figure 2.

**Figure 2:**
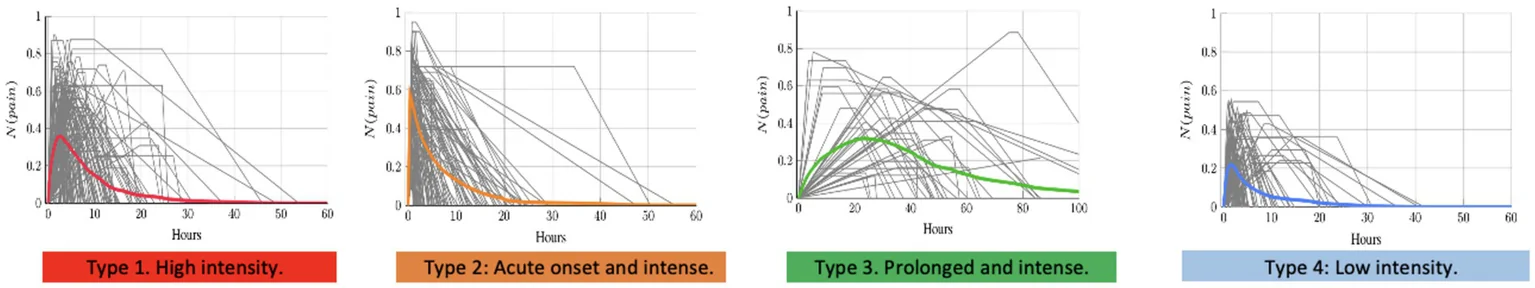
Four distinct attack-level migraine pain curve patterns based on pain dynamics. Type 1 (red): high intensity; Type 2 (orange): acute onset and intense; Type 3 (green): prolonged and intense; Type 4 (blue): low intensity. Modified from (9).

No single model-selection criterion identified a uniquely optimal cluster number. The permutation-based validation indicated that the clustering structure was statistically meaningful for k = 5, 6, and 9 (see Supplementary Table S1), whereas higher values yielded solutions that were not distinguishable from random partitioning. We therefore retained k = 5 as the primary solution because it provided the strongest balance between internal separation, bootstrap stability, and clinically interpretable subgroup structure. All clustering analyses were performed at the patient level (*N* = 51).

The WCSS curve showed a geometric elbow at k = 5 (Figure 3). For k = 5, the WCSS was 8.9, the Silhouette score was 0.447, the Calinski-Harabasz index was 32.397, and the Davies-Bouldin index was 0.821 as shown in Supplementary Table S1. Considered individually, Silhouette and Calinski-Harabasz are better at higher, less interpretable partitions (k = 6, 0.468, and k = 9, 35.667, respectively, both statistically significant against a permutation null), while the nominal Davies-Bouldin minimum (k = 9, 0.663) was not statistically significant (*p* = 0.069); the lowest Davies-Bouldin value reaching significance occurred at k = 8 (0.712). In the 100 patient-level bootstrap refits ARI was 0.831 ± 0.119. These values support k = 5 as a defensible compromise among separation, stability, cluster size, and interpretability, rather than as the uniquely optimal solution under every model-selection criterion. The GMM also supports k = 5, while hierarchical clustering varies from k = 4, 5, 6, and 10. Thus, as a stable value we keep k = 5 as the primary solution (see Supplementary Tables S2–S4 in Supplementary material).

**Figure 3:**
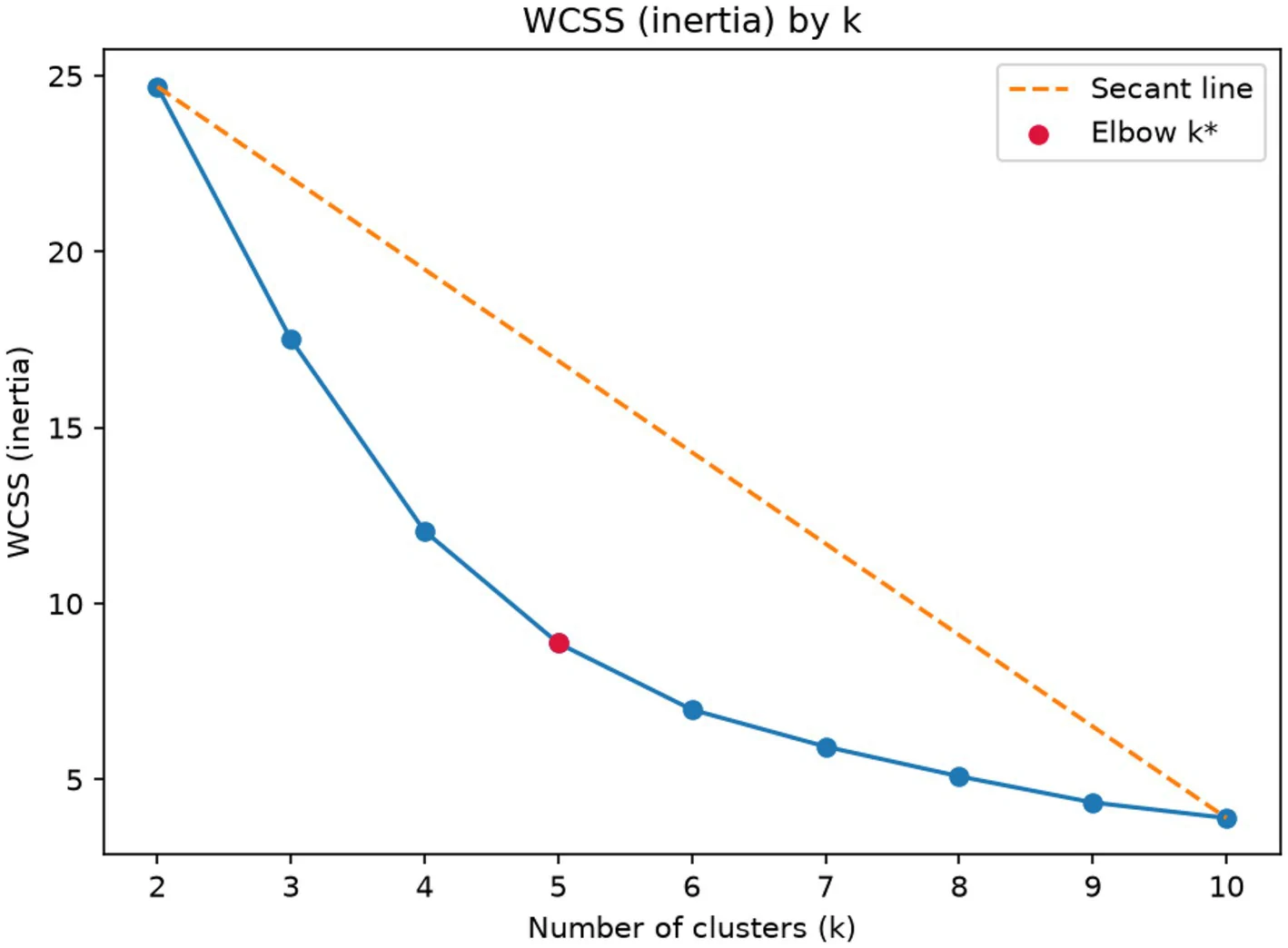
WCSS for k = 2–10. The dashed line indicates the secant joining the first and last evaluated k values. The selected elbow corresponds to k = 5, which was retained as the reference-guided exploratory solution.

Clusters were labeled according to the dominant attack-type proportion at the cluster centroid, resulting in five profiles: high-intensity (Group A), mixed pattern (Group B), acute-onset (Group C), prolonged-intense (Group D), and low-intensity (Group E). The emergence of five patient clusters despite the presence of four attack-level trajectory types reflects the heterogeneous combination of these patterns within individual patients. The internal composition of each cluster, expressed as the proportional distribution of attack types, is summarized in Table 2.

**Table 2:** Distribution of the types of migraine crises within each cohort of patients.

Migraine type / Patient cohort	A	B	C	D	E	Migraines per type
High intensity	87,1%	45,4%	17,5%	21,4%	3,8%	34,3%
Acute onset and intense	3,2%	23,0%	71,3%	14,3%	18,9%	31,1%
Prolonged and intense	0,0%	5,9%	0,0%	64,3%	11,3%	9,6%
Low intensity	9,7%	25,7%	11,3%	0,0%	66,0%	25,0%
Migraines per cohort	9,0%	44,2%	23,3%	8,1%	15,4%	100%

Following patient allocation, an uneven distribution across subgroups was observed. Group B was the most prevalent subgroup (20 patients, 39.2%), whereas Group D was the least represented (5 patients, 9.8%). The remaining groups included 7 patients in Group A (13.7%), 12 in Group C (23.5%), and 7 in Group E (13.7%). Because clustering was performed at the patient level, these 51 patients, rather than the 344 recorded attacks, represent the effective sample size for the classification analysis.

The distributions shown in Table 2 define the distinct clinical phenotypes of each cohort. Group B, the largest subgroup, exhibited a mixed clinical pattern in which no single migraine type predominated. High-intensity (45.4%) and low-intensity attacks (25.7%) were both frequent, while prolonged attacks were uncommon (5.9%).

The remaining cohorts showed more polarized clinical profiles. Group A was characterized by a highly homogeneous pattern, with high-intensity attacks accounting for 87.1% of episodes. Group C was predominantly defined by acute-onset attacks (71.3%). Group D (n = 5), should therefore be interpreted cautiously because its size limits the precision and reproducibility of cluster-specific estimates; however, despite being the smallest subgroup and contributing the lowest proportion of total recorded crises (8.1%), it was strongly characterized by prolonged attacks (64.3%). In contrast, Group E showed a clear predominance of low-intensity episodes (66.0%).

As a compositional-data sensitivity analysis, the four patient-level proportions were CLR-transformed after adding a pseudocount of 10^−6^ to zero proportions. CLR-based K-means was evaluated for k = 2–10 (Supplementary Table S5). The WCSS elbow again selected k = 5, but Silhouette, Calinski-Harabasz, and Davies-Bouldin were all optimal at k = 10, which is not clinically interpretable. This confirms that k = 5 is elbow-consistent but not universally optimal across validation criteria, so it is reported as a sensitivity analysis and does not replace the primary Euclidean solution.

### 3.3 Clinical characteristics across patient subgroups

No significant associations were found between patient subgroups and age, sex, age at migraine onset, cardiovascular risk factors, smoking status, family history of migraine, or baseline diagnosis. Preventive treatment use did not differ significantly across groups.

Table 3 presents the distribution of key clinical and sociodemographic variables across patient cohorts. Given the overall sample size (*N* = 51) and the resulting data sparsity in multi-level cross-tabulations, an exploratory regrouping to reduce sparse cells of both categories and cohorts was performed. This approach was used to reduce sparse or zero cells in patient-level contingency tables and to allow the use of exact or descriptive comparisons appropriate for the available sample size. However, categorizing variables and aggregating cohorts involved loss of information and did not increase the underlying sample size or independent statistical power. These analytical choices were therefore considered exploratory and were interpreted cautiously. The exploratory groupings evaluated in Table 4 are reported together with their corresponding uncorrected and Fisher exact *p*-values to rigorously account for small sample sizes.

**Table 3:** Distribution of key clinical variables across patient cohorts.

Cohort	Educational level		Cognitive reserve		Monthly number of migraines			
	Primary/Lower	University	≤14	>14	<1	1–8	9–14	≥14
A	5 (83.3%)	1 (16.7%)	4 (66.7%)	2 (33.3%)	0 (0.0%)	6 (100%)	0 (0.0%)	0 (0.0%)
B	4 (20.0%)	16 (80.0%)	4 (20.0%)	16 (80.0%)	1 (5.3%)	16 (84.2%)	1 (5.3%)	1 (5.3%)
C	1 (9.1%)	10 (90.9%)	2 (16.7%)	10 (83.3%)	2 (16.7%)	8 (66.7%)	2 (16.7%)	0 (0.0%)
D	1 (20.0%)	4 (80.0%)	2 (40.0%)	3 (60.0%)	0 (0.0%)	4 (80.0%)	1 (20.0%)	0 (0.0%)
E	1 (14.3%)	6 (85.7%)	1 (14.3%)	6 (85.7%)	0 (0.0%)	6 (85.7%)	0 (0.0%)	1 (14.3%)

**Table 4:** Comparative analysis of patient cohorts and sociodemographic variables.

Clinical variable	Categorization	Cohort grouping	*p*-value (FDR significant)	Fisher’s exact *p*-value (FDR significant)
Educational level	Non-university vs. university	None (A, B, C, D, E)	0.010 (Yes)	0.015 (Yes)
Cognitive reserve	≤14 vs. > 14	None (A, B, C, D, E)	0.132 (−)	-
		A-D vs. B-C-E	0.014 (Yes)	0.023 (Yes)
Migraines per month	<1, 1–8, 9–14, ≥14	None (A, B, C, D, E)	0.124 (−)	- (−)
	1–8 vs. Rest		0.030 (No)	0.581 (−)
Smoker	No vs. yes	A-B-E vs. C-D	0.063 (−)	- (−)
Age of onset	<10 vs. 10–39	A-B-C-D vs. E	0.064 (−)	- (−)
Diagnosis	Without aura vs. rest	A vs. B-C-D-E	0.082 (−)	- (−)
Family history	No vs. yes	A-E vs. B vs. C-D	0.125 (−)	- (−)
Age	18–39 vs. 40–65	A-C-D-E vs. B	0.160 (−)	- (−)
Sex	Male vs. female	None (A, B, C, D, E)	0.586 (−)	- (−)
Dyslipidemia	No vs. yes	A-D-E vs. B vs. C	0.239 (−)	- (−)
Cardiovascular risk	No vs. yes	A-C-D-E vs. B	0.248 (−)	- (−)

Educational level, categorized as non-university versus university education, showed an exploratory association with the five patient cohorts (Fisher’s exact test, *p* = 0.015), which remained below the prespecified threshold after FDR correction. Given the small and imbalanced sample and the categorization of the variable, this finding should be interpreted cautiously.

Cognitive reserve, stratified using a 14-point threshold, was explored using the A-D versus B-C-E cohort grouping. This downstream exploratory regrouping was selected to reduce sparse cells in the patient-level contingency table and was not used to define the original five cohorts. The exact test yielded *p* = 0.023, which remained below the prespecified threshold after FDR correction. This result should also be interpreted cautiously given the exploratory regrouping and the small, unequal cohort sizes.

Migraine attack frequency per month did not differ significantly across the five patient cohorts. Given the small number of patients in some frequency categories, an exploratory patient-level analysis was performed comparing patients with 1–8 attacks per month with those in the remaining frequency categories. Although the uncorrected asymptotic test suggested a potential association, this finding was not confirmed by Fisher’s exact test (*p* = 0.582). Therefore, no significant association between monthly migraine attack frequency and patient cohort was identified.

Given the exploratory nature of the study, all analyzed variables are reported to provide a comprehensive description of their distribution across the five patient cohorts. Associations not retained after exact testing or FDR correction are interpreted as exploratory.

While the initial patient-level analysis did not reveal a statistically significant association regarding clinical diagnosis (migraine without aura versus other subtypes), the proportion of attacks with aura varied across cohorts at the attack level (Table 5), being highest in Groups E (26.4%) and D (17.9%) and below 10% in the remaining subgroups. This descriptive comparison was statistically significant (Fisher’s exact test, *p* < 0.001), but it should be interpreted cautiously because the attack-level analysis includes 344 observations nested within the same 51 patients. Although the attack-level dataset contains more records, these observations are not statistically independent and therefore do not represent a proportional increase in effective sample size or statistical power. This is particularly relevant for aura, which is largely a patient-level characteristic; the 34 attacks with aura were contributed by a small number of patients. When this dependence was formally accounted for using a generalized estimating equation with patient-level clustering, the association was no longer statistically significant (*p* = 0.182). The attack-level aura distribution is therefore reported as a descriptive observation consistent with the patient-level analysis, rather than as an independent statistical finding.

**Table 5:** Distribution of migraine attacks with/ without aura by cohort.

Cohort	Migraines without aura (%)	Migraines with aura (%)
A	28 (90.3%)	3 (9.7%)
B	145 (95.4%)	7 (4.6%)
C	75 (93.8%)	5 (6.3%)
D	23 (82.1%)	5 (17.9%)
E	39 (73.6%)	14 (26.4%)

Values are expressed as *n* (%) of attacks within each cohort. Fisher’s exact test was used for the descriptive comparison of the observed attack distributions (*p* < 0.001). Logistic generalized estimating equation did not show a significant association (*p* = 0.182).

Table 6 shows pain intensity across attacks, supporting the consistency of the cluster nomenclature (Fisher’s exact test, *p* < 0.0001). Groups classified as “high intensity” (Group A) and “prolonged and intense” (Group D) showed a higher proportion of high-intensity scores, whereas the “low intensity” group (Group E) demonstrated predominantly lower intensity values, reinforcing the internal coherence of the classification.

**Table 6:** Distribution of migraine attacks intensity by cohort.

Patient cohort	≤5	6–7	8–10
A	1 (3.2%)	11 (35.5%)	19 (61.3%)
B	27 (17.8%)	76 (50.0%)	49 (32.2%)
C	15 (18.8%)	36 (45.0%)	29 (36.3%)
D	1 (3.6%)	11 (39.3%)	16 (57.1%)
E	40 (75.5%)	12 (22.6%)	1 (1.9%)

### 3.4 Secondary analysis of premonitory, concomitant and postdromic symptoms

#### 3.4.1 Premonitory symptoms according to patient cohort

When the premonitory symptom count was compared across the five patient cohorts, a significant global difference emerged under the unclustered one-way ANOVA (*p* < 0.001) (Table 7). The patient-clustered Poisson GEE also showed a significant global difference across cohorts (Wald *p* = 0.010). *Post hoc* pairwise comparisons with Benjamini-Hochberg correction showed that cohort E had significantly fewer premonitory symptoms per attack than cohorts A, B, and D. The corresponding FDR-adjusted *p*-values from the Poisson GEE were 0.008, 0.008, and 0.028, respectively; no other pairwise comparison remained significant after correction.

**Table 7:** Premonitory symptom count by patient cohort.

Patient cohort	Premonitories mean ± std
A (*n* = 7)	4.32 ± 4.17
B (*n* = 20)	3.46 ± 3.88
C (*n* = 12)	2.73 ± 2.93
D (*n* = 5)	3.57 ± 3.80
E (*n* = 7)	0.94 ± 1.98

Global one-way ANOVA (p < 0.001) and patient-clustered Poisson GEE global Wald test (p = 0.010). Pairwise comparisons were adjusted using the Benjamini-Hochberg procedure.

Individual premonitory symptoms were explored across attacks contributed by the five patient cohorts. Because several symptoms were infrequent and some cohorts were small, reliable GEE estimates could not be obtained for 17 of the 33 symptoms due to quasi-complete separation; these results were therefore reported as not estimable (NE). Among the 16 symptoms for which clustered GEE models could be reliably estimated, neck stiffness was the only individual premonitory symptom that remained significantly associated with the patient cohort after FDR correction (GEE-FDR *p* = 0.0003). (Supplementary Table S6). Neck stiffness was most frequently recorded in attacks from patients in the high-intensity profile (Group A, 54.8%) and prolonged-intense profile (Group D, 39.3%), and least frequently in the acute-onset profile (Group C, 6.2%). Although several other symptoms showed differences in descriptive Fisher analyses, these associations were not confirmed after accounting for within-patient clustering using Poisson GEE for the total symptom count and logistic GEE for individual binary symptoms. These individual symptom findings should therefore be considered exploratory.

#### 3.4.2 Concomitant symptoms according to patient cohort

The overall burden of concomitant symptoms differed significantly across the five patient cohorts after accounting for within-patient clustering (GEE *p* = 0.0057). Group D showed the highest mean concomitant symptom burden (2.89 ± 1.45 symptoms per attack), closely followed by Group A (2.87 ± 1.43), whereas Group B showed the lowest mean burden (1.93 ± 1.27). After FDR correction for pairwise comparisons, only Group A showed a significantly higher concomitant symptom burden than Group E (*p* = 0.0353; Table 8).

**Table 8:** Concomitant symptom burden according to patient cohort.

Cohort	Mean ± SD	*n*
A	2.87 ± 1.43	31
B	1.93 ± 1.27	152
C	2.09 ± 1.47	80
D	2.89 ± 1.45	28
E	2.09 ± 1.06	53

Values are expressed as mean ± standard deviation (SD) of the number of concomitant symptoms recorded per migraine attack. *n* represents the number of recorded attacks within each patient cohort. Global differences between cohorts were assessed using a generalized estimating equation (GEE) accounting for within-patient clustering (*p* = 0.0057). Pairwise comparisons were corrected for multiple testing using the Benjamini-Hochberg false discovery rate (FDR) procedure.

Concomitant symptoms also differed across the five patient profiles. After accounting for within-patient clustering using GEE and correcting for multiple comparisons, significant differences across cohorts remained for nausea (FDR-adjusted *p* = 0.0162), photophobia (*p* = 0.0162), osmophobia (*p* = 0.0162), and other concomitant symptoms (*p* = 0.0045). (Supplementary Table S7). Nausea was most frequent in the prolonged-intense profile (Group D, 82.1%), photophobia was particularly frequent in the low-intensity (Group E, 90.6%) and high-intensity (Group A, 87.1%) profiles, and osmophobia was most frequent in Groups D (50.0%) and A (48.4%). In contrast, vomiting and phonophobia did not differ significantly across patient profiles after correction.

#### 3.4.3 Postdromic symptoms according to patient cohort

When the postdromic symptom count was compared across the five patient cohorts, a significant global difference was observed under the unclustered one-way ANOVA (*p* < 0.001; Table 9). The patient-clustered Poisson GEE also showed a significant global difference across cohorts (Wald *p* = 0.029). *Post hoc* pairwise comparisons with Benjamini-Hochberg correction showed that cohort D had a significantly higher postdromic symptom burden than cohort E (GEE FDR-adjusted *p* = 0.044), while no other pairwise comparison remained significant after correction.

**Table 9:** Postdromic symptom count by patient cohort.

Patient cohort	Postdromic mean ± std
A (*n* = 7)	2.32 ± 1.90
B (*n* = 20)	2.35 ± 1.70
C (*n* = 12)	1.66 ± 1.53
D (*n* = 5)	3.39 ± 1.87
E (*n* = 7)	1.92 ± 1.45

Global one-way ANOVA (*p* < 0.001) and patient-clustered Poisson GEE global Wald test (*p* = 0.029). Pairwise comparisons were adjusted using the Benjamini-Hochberg procedure; only the comparison between cohorts D and E remained significant (FDR-adjusted *p* = 0.044).

Individual postdromic symptoms showed descriptive differences across the five patient cohorts. Appetite changes, food craving, cognitive symptoms, temperature changes, and yawning differed significantly in the unadjusted Fisher analyses. However, none of these associations remained significant after accounting for within-patient clustering using GEE and correcting for multiple comparisons. Therefore, no individual postdromic symptom was robustly associated with patient cohort (Supplementary Table S8).

### 3.5 Exploratory sensitivity analysis of acute treatment across patient profiles

Acute treatment was explored as a potential modifier of pain-trajectory profiles. After accounting for repeated attacks within patients, no robust differences were observed across the five patient profiles in the type of rescue medication used or in the use of any rescue medication. The number of acute medications per attack also did not differ significantly across profiles (Poisson GEE *p* = 0.454). In contrast, the timing of the first acute medication relative to the pain peak differed significantly across profiles (Gaussian GEE *p* = 0.000020). The prolonged-intense profile (Group D) showed the latest treatment timing, with the first medication recorded a mean of 19.9 ± 28.3 h relative to the pain peak, compared with 6.6 ± 23.2 h in Group A, 2.2 ± 18.2 h in Group B, 0.3 ± 2.3 h in Group C, and 3.1 ± 13.1 h in Group E. After FDR correction, treatment was significantly later in Group D than in each of the other four profiles, with model-estimated pairwise differences ranging from 13.6 to 18.7 h (all adjusted *p* < 0.05). These findings indicate that treatment timing, rather than medication type or amount, was the main acute-treatment feature associated with patient profile, with the detailed results of this exploratory sensitivity analysis provided in Supplementary Table S9.

## 4 Discussion

Data-driven phenotyping and machine-learning approaches have increasingly been explored in headache disorders, including cluster-based identification of clinical subphenotypes and decision-support models (11, 12).

Within this data-driven framework, our study introduces a novel approach to classifying migraine patients based on pain curve dynamics, contributing to the evolving landscape of data-driven and digital phenotyping in migraine research (13, 14). Building upon previously identified attack-level pain trajectories, we demonstrate that these dynamic patterns translate into five distinct patient-level profiles: high-intensity (Group A), mixed pattern (Group B), acute-onset (Group C), prolonged-intense (Group D), and low-intensity (Group E). Notably, the mixed profile was the most frequent, whereas the remaining profiles showed a clearer predominance of a specific attack trajectory. This finding highlights the substantial intra-individual variability of migraine, as patients may experience different types of attacks rather than consistently expressing a single pain trajectory. This stratification reinforces the heterogeneity of migraine expression and supports the concept that patients may cluster according to predominant pain evolution patterns rather than solely traditional diagnostic criteria (15, 16). These findings may be relevant to the emerging paradigm of precision neurology, in which disease phenotyping increasingly incorporates high-resolution longitudinal data.

Importantly, demographic and most baseline clinical variables, including age, sex, baseline diagnosis, and monthly migraine attack frequency, did not differ significantly across subgroups, suggesting that dynamic pain profiles are not simply reflections of traditional clinical descriptors. Instead, they appear to represent an additional layer of phenotypic variability within episodic migraine. The observed differences in educational level and cognitive reserve may reflect additional variability among patient profiles, although these associations should be interpreted cautiously and considered exploratory and hypothesis-generating. Their potential clinical relevance remains to be confirmed in larger, independent cohorts.

The aura distribution showed a differential descriptive pattern, with a higher proportion of attacks with aura in the low-intensity (Group E) and prolonged-intense (Group D) profiles, while remaining below 10% in the other groups. However, this association was not maintained after accounting for the clustering of attacks within patients and should therefore be considered exploratory. Nevertheless, the presence of aura in profiles with markedly different pain characteristics may suggest that aura expression is not directly related to pain intensity. This would be consistent with the possibility that mechanisms related to aura, including cortical spreading depression, represent a partially independent dimension of migraine heterogeneity.

The internal coherence of the classification was supported by reported pain intensity. High-intensity and prolonged-intense profiles consistently showed higher pain scores, whereas the low-intensity profile showed lower values, reinforcing that the clusters reflect meaningful differences in clinical expression rather than computational artifacts.

Beyond pain intensity, the five patient profiles also showed differences in the clinical expression of migraine across different phases of the attack. During the headache phase, the overall burden of concomitant symptoms differed across profiles after accounting for within-patient clustering, with the high-intensity profile (Group A) showing a greater burden than the low-intensity profile (Group E) after FDR correction. Individual concomitant symptoms also varied across patient profiles, with significant differences for nausea, photophobia, and osmophobia after adjustment for within-patient dependence and multiple comparisons. Nausea was particularly frequent in the prolonged-intense profile (Group D), photophobia in the high- and low-intensity profiles (Groups A and E), and osmophobia in the high-intensity and prolonged-intense profiles (Groups A and D). Differences were also observed before and after the headache phase. The low-intensity profile (Group E) showed a lower premonitory symptom burden than the high-intensity (Group A), mixed (Group B), and prolonged-intense (Group D) profiles. Among individual premonitory symptoms, neck stiffness was the only symptom that remained significantly associated with patient cohort after accounting for within-patient clustering and multiple comparisons. In the postdromic phase, the prolonged-intense profile (Group D) showed a greater overall symptom burden than the low-intensity profile (Group E), although no individual postdromic symptom remained significantly associated with patient cohort after clustered analysis and FDR correction. The lower premonitory symptom burden observed in Group E is consistent with previous evidence linking a greater number of premonitory symptoms to more severe migraine, including higher headache intensity and longer attack duration (17). From a pathophysiological perspective, these findings may suggest that the identified patient profiles capture differences in the broader expression of the migraine cycle rather than merely differences in pain intensity or duration. The greater postdromic burden in the prolonged-intense profile may similarly reflect a more prolonged recovery following the headache phase. Overall, these findings support the clinical relevance of patient stratification based on pain dynamics and suggest that the resulting profiles may represent broader migraine phenotypes encompassing the premonitory, headache, and postdromic phases.

Acute treatment represents a potentially relevant modifier of pain evolution and was therefore explored as a sensitivity analysis. Reassuringly, patient profiles were not associated with robust differences in the type of rescue medication used, overall rescue-medication use, or the number of acute medications per attack after accounting for repeated attacks within patients. This suggests that the identified profiles are unlikely to simply reflect differences in acute pharmacological exposure. However, treatment timing differed substantially across profiles, with patients in the prolonged-intense profile recording their first acute medication significantly later than patients in all other profiles. This finding may be clinically relevant, as treatment timing could influence pain evolution and attack duration. Conversely, a prolonged or intense attack trajectory could itself influence when treatment is taken. Given the observational design, the direction of this relationship cannot be established, and treatment timing should therefore be considered a potential modifier of pain trajectory rather than a causal explanation for the identified profiles.

By demonstrating that dynamic pain trajectories are associated with differences in premonitory and postdromic symptom burden, as well as with a differential descriptive pattern in aura distribution, our findings align with emerging studies emphasizing the importance of detailed symptom tracking in migraine management (4, 18). Identifying pain curve dynamics as a basis for patient stratification reflects a growing trend in applying machine learning techniques to medical data, which may contribute to more individualized treatment strategies (16, 19). Rather than relying exclusively on traditional diagnostic descriptors, this approach captures clinically meaningful variability in pain evolution patterns, reinforcing the complexity and diversity of migraine presentations (20, 21).

From a clinical perspective, these dynamic profiles may contribute in the future to a more precise characterization of patients and help guide therapeutic decision-making. The differences observed across profiles provide a basis for exploring whether this stratification may be associated with treatment response, prognosis, or other clinically relevant outcomes. Although these potential applications will need to be confirmed in prospective studies, the current findings support further evaluation of this approach within the context of personalized medicine (15, 22, 23). If validated, this classification could also be useful for clinical trial design by enabling the identification of more homogeneous patient subgroups according to their attack dynamics.

In the future, this approach could be extended to other neurological disorders characterized by episodic symptoms that vary over time, such as epilepsy (24–27). In migraine, it will be important to validate this patient-level classification in larger and more diverse cohorts, as well as to explore the potential biological and physiological correlates of these profiles (28).

In summary, this study expands current migraine classification approaches by demonstrating that attack-level pain dynamics can meaningfully stratify patients into clinically distinguishable profiles. By integrating real-time digital symptom capture with data-driven clustering techniques, this framework offers a structured and reproducible method to better understand migraine heterogeneity and may contribute to more tailored management strategies.

### 4.1 Strengths and limitations

A major strength of this study is its longitudinal design, with prospective, real-time recording of multiple migraine attacks per patient. This approach reduced recall bias, captured intra-individual variability, and provided a richer characterization of migraine dynamics than would be possible from a single cross-sectional assessment.

Several limitations should be considered. The sample was relatively small (51 patients), was recruited from a single center, and the five patient clusters were imbalanced (7, 20, 12, 5, and 7 patients), with only five patients in Group D. Although 344 attacks were recorded, these were nested within the 51 participants and therefore did not increase the effective sample size for patient-level clustering. The underrepresentation of men also limits sex-specific conclusions. Nevertheless, the prospective assessment of multiple attacks per patient provided substantial longitudinal information and enabled the characterization of patterns that may not be apparent from conventional baseline variables alone.

The requirement of at least three recorded attacks may have favored the inclusion of patients with higher attack frequency or better adherence to prospective recording, and the 4–6 week observation period was insufficient to establish long-term profile stability. However, this period allowed repeated characterization of individual attacks and provided the basis for identifying consistent within-patient patterns.

The clustering approach also assumes that the predefined attack-level trajectories adequately capture the complexity of migraine pain evolution. Nevertheless, the five-profile solution showed good internal reproducibility in bootstrap analyses, although it was not uniformly optimal across all clustering methods. Overall, these findings support the clinical interpretability and internal consistency of the proposed classification while highlighting the need for external validation in larger independent cohorts.

Several continuous variables were categorized to reduce sparse cells across the five unequal cohorts, which may have reduced statistical resolution and obscured potential dose–response relationships. In addition, multiple exploratory subgroup and symptom comparisons increase the possibility of chance findings despite FDR correction. Therefore, the associations observed with educational level and cognitive reserve should be considered hypothesis-generating, while potentially providing relevant signals for future research.

The study primarily focused on pain trajectories and did not systematically assess genetic or environmental factors, additional comorbidities, attack triggers, or menstrual-cycle effects. Although acute treatment was explored in a sensitivity analysis, residual confounding related to treatment timing and its potential influence on pain evolution and attack duration cannot be excluded. Treatment response, disability outcomes, and long-term prognosis were also not evaluated.

Overall, the prospective characterization of multiple attacks within individual patients provides a robust framework for exploring migraine heterogeneity at the patient level. Despite the limitations inherent to an exploratory single-center study, these findings support the potential value of temporal pain dynamics for dynamic migraine phenotyping and provide a clear rationale for validation in larger, more diverse independent cohorts with longitudinal follow-up.

## 5 Conclusion

This study supports the use of pain trajectory dynamics as a meaningful basis for stratifying patients with migraine. The combination of four previously identified attack-level pain trajectories allowed the definition of five distinct patient profiles: a mixed profile, which was the most frequent, and other profiles characterized by the clear predominance of a specific attack type.

These profiles were not explained by most conventional demographic or clinical variables, but were associated with distinct patterns of symptom expression throughout the migraine attack, including differences in associated symptom burden and in symptoms occurring before and after the headache phase.

Overall, these findings provide a proof of concept for dynamic patient phenotyping based on pain evolution and associated clinical features, beyond traditional diagnostic classifications. Further validation in larger, independent cohorts will be necessary to confirm the robustness and clinical applicability of this classification.

## Data Availability

The raw data supporting the conclusions of this article will be made available by the authors upon reasonable request.
