# Supplementary material for: Patient classification in episodic migraine based on data-driven pain curve dynamics
Source: Front Neurol. 2026 Oct 13;17:1853701. doi: 10.3389/fneur.2026.1853701 (PMC13710035; doi:10.3389/fneur.2026.1853701)
Supplement: Supplementary file 1 [file Table_1.docx]

***Supplementary Table S1.*** *Clustering quality metrics for K-means solutions with 2-10 clusters. Bootstrap ARI values are presented across 100 patient-level bootstrap resamples. WCSS and Silhouette p-values are empirical p-values obtained from 100 column-wise permutation tests. Higher Silhouette and Calinski-Harabasz values and lower Davies-Bouldin values indicate better internal separation; WCSS is expected to decrease as k increases.*

| **k** | **WCSS** | **WCSS p-value** | **Silhouette** | **Silhouette p-value** | **Calinski- Harabasz** | **Calinski- Harabasz p-value** | **Davies Bouldin** | **Davies Bouldin p-value** | **Bootstrap ARI mean ± std** |
| --- | --- | --- | --- | --- | --- | --- | --- | --- | --- |
| **2** | 24.7 | 0.168 | 0.285 | 0.089 | 18.234 | 0.168 | 1.542 | 0.257 | 0.348 ± 0.283 |
| **3** | 17.5 | 0.010 | 0.357 | 0.010 | 22.447 | 0.010 | 1.166 | 0.050 | 0.562 ± 0.244 |
| **4** | 12.1 | 0.010 | 0.415 | 0.010 | 28.348 | 0.010 | 0.914 | 0.010 | 0.747 ± 0.159 |
| **5** | **8.9** | **0.010** | 0.448 | 0.010 | 32.397 | 0.010 | 0.821 | 0.010 | 0.831 ± 0.119 |
| **6** | 7.0 | 0.010 | **0.468** | **0.020** | 34.646 | 0.010 | 0.813 | 0.020 | **0.838 ± 0.101** |
| **7** | 5.9 | 0.010 | 0.434 | 0.069 | 34.521 | 0.010 | 0.820 | 0.168 | 0.772 ±0.092 |
| **8** | 5.1 | 0.010 | 0.437 | 0.149 | 34.736 | 0.010 | **0.712** | **0.030** | 0.758 ±0.105 |
| **9** | 4.3 | 0.020 | 0.445 | 0.168 | **35.667** | **0.020** | 0.663 | 0.069 | 0.730 ±0.105 |
| **10** | 3.9 | 0.040 | 0.437 | 0.356 | 34.944 | 0.040 | 0.665 | 0.079 | 0.701 ±0.102 |

***Supplementary Table S2.*** *Ward hierarchical clustering sensitivity analysis for k=2-10. WCSS, Silhouette, Calinski-Harabasz, Davies-Bouldin, and intragroup variance relative to the corresponding K-means solution are reported. The maximum Silhouette was observed at k=4, the minimum Davies-Bouldin index at k=6, and the maximum Calinski-Harabasz value at k=10. WCSS was used descriptively and not as a standalone criterion for selecting k.*

| **k** | **WCSS** | **Silhouette** | **Calinski-Harabasz** | **Davies Bouldin** | **Intragroup variance** |
| --- | --- | --- | --- | --- | --- |
| **2** | 11.2 | 0.293 | 19.867 | 1.026 | 11.197 |
| **3** | 7.6 | 0.341 | 26.022 | 1.096 | 7.551 |
| **4** | 4.7 | **0.417** | 37.261 | 0.824 | 4.658 |
| **5** | **3.5** | **0.417** | 39.522 | 0.765 | 3.547 |
| **6** | 3.0 | 0.408 | 38.633 | **0.760** | 2.973 |
| **7** | 2.5 | 0.401 | 38.788 | 0.789 | 2.502 |
| **8** | 2.1 | 0.414 | 40.262 | 0.797 | 2.083 |
| **9** | 1.7 | 0.385 | 44.245 | 0.808 | 1.669 |
| **10** | 1.5 | 0.402 | **44.293** | 0.781 | 1.468 |

***Supplementary Table S3.*** *Complete-linkage hierarchical clustering sensitivity analysis for k=2-10. WCSS, Silhouette, Calinski-Harabasz, Davies-Bouldin, and intragroup variance relative to the corresponding K-means solution are reported. The maximum Silhouette, maximum Calinski-Harabasz value, and minimum Davies-Bouldin index were all observed at k=10. WCSS was used descriptively and not as a standalone criterion for selecting k.*

| **k** | **WCSS** | **Silhouette** | **Calinski-Harabasz** | **Davies Bouldin** | **Intragroup variance** |
| --- | --- | --- | --- | --- | --- |
| **2** | 11.5 | 0.237 | 18.057 | 1.314 | 11.500 |
| **3** | 9.3 | 0.265 | 16.469 | 1.522 | 9.333 |
| **4** | 6.5 | 0.323 | 22.275 | 0.897 | 6.498 |
| **5** | **4.3** | 0.332 | 30.581 | 0.888 | 4.301 |
| **6** | 3.4 | **0.340** | 32.271 | 0.850 | 3.432 |
| **7** | 2.7 | 0.337 | 35.088 | 0.885 | 2.720 |
| **8** | 2.3 | 0.354 | 36.467 | 0.800 | 2.269 |
| **9** | 2.1 | 0.363 | 34.698 | 0.758 | 2.068 |
| **10** | 1.5 | 0.406 | **43.411** | **0.753** | 1.495 |

***Supplementary Table S4.*** *Gaussian mixture model sensitivity analysis for k=2-10. WCSS, Silhouette, Calinski-Harabasz, Davies-Bouldin, and BIC are reported. WCSS was used descriptively, whereas BIC was used as the primary criterion for selecting the number of mixture components; the minimum BIC was obtained at k=5.*

| **k** | **WCSS** | **Silhouette** | **Calinski-Harabasz** | **Davies Bouldin** | **Intragroup Variance** | **BIC** |
| --- | --- | --- | --- | --- | --- | --- |
| **2** | 13.2 | 0.194 | 9.581 | 1.543 | 13.163 | -862.4 |
| **3** | 10.9 | 0.217 | 10.500 | 1.696 | 10.948 | -944.1 |
| **4** | 9.4 | 0.102 | 10.438 | 1.466 | 9.445 | -975.5 |
| **5** | 6.8 | 0.130 | 15.093 | 1.250 | 6.805 | **-981.0** |
| **6** | 7.0 | 0.094 | 11.251 | 1.375 | 6.994 | -955.9 |
| **7** | 8.0 | -0.019 | 7.107 | 1.890 | 7.992 | -954.5 |
| **8** | 5.6 | 0.141 | 11.147 | 1.272 | 5.591 | -910.4 |
| **9** | 5.0 | 0.098 | 11.196 | 0.971 | 5.024 | -830.2 |
| **10** | 3.1 | 0.202 | 18.779 | 1.288 | 3.072 | -836.4 |

***Supplementary Table S5.*** *CLR sensitivity analysis, K-means solutions with 2-10 clusters (N=51). p-values are empirical from 100 permutations; ARI is bootstrap-based (100 resamples). Again k=5 marks the geometric WCSS elbow.*

| **k** | **WCSS** | **WCSS p-value** | **Silhouette** | **Silhouette p-value** | **Calinski- Harabasz** | **Calinski- Harabasz p-value** | **Davies Bouldin** | **Davies Bouldin p-value** | **Bootstrap ARI mean ± std** |
| --- | --- | --- | --- | --- | --- | --- | --- | --- | --- |
| **2** | 3395.4 | 0.010 | 0.384 | 0.010 | 27.425 | 0.010 | 1.206 | 0.010 | 0.661 ± 0.300 |
| **3** | 2495.8 | 0.010 | 0.431 | 0.010 | 26.925 | 0.010 | 1.123 | 0.010 | 0.621 ± 0.241 |
| **4** | 1856.2 | 0.010 | 0.477 | 0.010 | 29.030 | 0.010 | 0.930 | 0.010 | 0.665 ± 0.215 |
| **5** | **1340.8** | 0.010 | 0.556 | 0.010 | 33.922 | 0.010 | 0.826 | 0.010 | 0.802 ± 0.144 |
| **6** | 1084.7 | 0.010 | 0.616 | 0.010 | 34.939 | 0.010 | 0.746 | 0.010 | 0.783 ± 0.078 |
| **7** | 865.2 | 0.010 | 0.656 | 0.010 | 37.553 | 0.010 | 0.750 | 0.010 | 0.839 ± 0.067 |
| **8** | 632.6 | 0.010 | 0.716 | 0.010 | 45.280 | 0.010 | 0.737 | 0.010 | 0.863 ± 0.052 |
| **9** | 424.5 | 0.010 | 0.774 | 0.010 | 60.252 | 0.010 | 0.608 | 0.010 | 0.919 ± 0.059 |
| **10** | 295.3 | 0.010 | **0.821** | **0.010** | **77.154** | **0.010** | **0.479** | **0.010** | **0.934 ± 0.065** |

*Supplementary Table S6.* ***Distribution of premonitory symptoms across patient cohorts.****Values are expressed as n (%) of attacks. Group A: high-intensity; Group B: mixed pattern; Group C: acute-onset; Group D: prolonged-intense; Group E: low-intensity. GEE analyses accounted for within-patient clustering, and p-values were FDR-adjusted. NE, not estimable due to sparse data or quasi-complete separation.*

| **Symptom** | **A n (%)** | **B n (%)** | **C n (%)** | **D n (%)** | **E n (%)** | **Fisher FDR** | **GEE FDR** |
| --- | --- | --- | --- | --- | --- | --- | --- |
| **Sadness** | 3 (9.7%) | 17 (11.2%) | 8 (10.0%) | 1 (3.6%) | 1 (1.9%) | 0.3726 | 0.5147 |
| **Euphoria** | 0 (0.0%) | 2 (1.3%) | 0 (0.0%) | 0 (0.0%) | 0 (0.0%) | 0.7941 | NE |
| **Hopelessness** | 0 (0.0%) | 4 (2.6%) | 1 (1.2%) | 1 (3.6%) | 0 (0.0%) | 0.6368 | NE |
| **Anxiety** | 4 (12.9%) | 16 (10.5%) | 8 (10.0%) | 4 (14.3%) | 3 (5.7%) | 0.7094 | 0.9068 |
| **Irritability** | 4 (12.9%) | 22 (14.5%) | 11 (13.8%) | 4 (14.3%) | 2 (3.8%) | 0.3886 | 0.6066 |
| **Apathy** | 3 (9.7%) | 21 (13.8%) | 12 (15.0%) | 1 (3.6%) | 0 (0.0%) | 0.0345 | NE |
| **Increased appetite** | 0 (0.0%) | 14 (9.2%) | 2 (2.5%) | 2 (7.1%) | 2 (3.8%) | 0.2529 | NE |
| **Decreased appetite** | 2 (6.5%) | 11 (7.2%) | 1 (1.2%) | 1 (3.6%) | 0 (0.0%) | 0.1576 | NE |
| **Craving specific foods** | 1 (3.2%) | 13 (8.6%) | 3 (3.8%) | 3 (10.7%) | 1 (1.9%) | 0.3886 | 0.5147 |
| **Thirst** | 6 (19.4%) | 27 (17.8%) | 7 (8.8%) | 5 (17.9%) | 1 (1.9%) | 0.0345 | 0.5147 |
| **Insomnia** | 4 (12.9%) | 7 (4.6%) | 4 (5.0%) | 2 (7.1%) | 2 (3.8%) | 0.4847 | 0.5147 |
| **Drowsiness** | 9 (29.0%) | 45 (29.6%) | 13 (16.2%) | 3 (10.7%) | 3 (5.7%) | **0.0043** | 0.1745 |
| **Difficulty concentrating** | 4 (12.9%) | 32 (21.1%) | 16 (20.0%) | 4 (14.3%) | 5 (9.4%) | 0.4534 | 0.9411 |
| **Difficulty speaking** | 1 (3.2%) | 9 (5.9%) | 4 (5.0%) | 4 (14.3%) | 1 (1.9%) | 0.3886 | 0.5147 |
| **Difficulty writing** | 0 (0.0%) | 1 (0.7%) | 3 (3.8%) | 0 (0.0%) | 1 (1.9%) | 0.4735 | NE |
| **Feeling colder** | 2 (6.5%) | 10 (6.6%) | 6 (7.5%) | 0 (0.0%) | 1 (1.9%) | 0.5088 | NE |
| **Feeling hotter** | 1 (3.2%) | 10 (6.6%) | 6 (7.5%) | 4 (14.3%) | 0 (0.0%) | 0.1576 | NE |
| **Nausea and/or vomiting** | 3 (9.7%) | 22 (14.5%) | 9 (11.2%) | 3 (10.7%) | 0 (0.0%) | 0.0740 | NE |
| **Flatulence** | 1 (3.2%) | 11 (7.2%) | 0 (0.0%) | 2 (7.1%) | 2 (3.8%) | 0.1576 | NE |
| **Constipation** | 1 (3.2%) | 10 (6.6%) | 1 (1.2%) | 0 (0.0%) | 0 (0.0%) | 0.2044 | NE |
| **Fluid retention/swelling** | 1 (3.2%) | 10 (6.6%) | 1 (1.2%) | 1 (3.6%) | 2 (3.8%) | 0.5088 | 0.6953 |
| **Increased urination frequency** | 1 (3.2%) | 4 (2.6%) | 0 (0.0%) | 1 (3.6%) | 0 (0.0%) | 0.3917 | NE |
| **Neck stiffness** | 17 (54.8%) | 38 (25.0%) | 5 (6.2%) | 11 (39.3%) | 8 (15.1%) | **0.0003** | **0.0003** |
| **Yawning** | 10 (32.3%) | 38 (25.0%) | 10 (12.5%) | 4 (14.3%) | 2 (3.8%) | **0.0043** | 0.1745 |
| **Fatigue** | 14 (45.2%) | 22 (14.5%) | 15 (18.8%) | 5 (17.9%) | 3 (5.7%) | **0.0043** | 0.2919 |
| **Sensitivity to light (photophobia)** | 11 (35.5%) | 36 (23.7%) | 22 (27.5%) | 8 (28.6%) | 5 (9.4%) | 0.1100 | 0.6066 |
| **Sensitivity to sound (phonophobia)** | 6 (19.4%) | 17 (11.2%) | 17 (21.2%) | 5 (17.9%) | 3 (5.7%) | 0.1539 | 0.5147 |
| **Sensitivity to smells (osmophobia)** | 4 (12.9%) | 8 (5.3%) | 2 (2.5%) | 1 (3.6%) | 0 (0.0%) | 0.1576 | NE |
| **Sensitivity to touch (allodynia)** | 0 (0.0%) | 6 (3.9%) | 1 (1.2%) | 2 (7.1%) | 0 (0.0%) | 0.3726 | NE |
| **Ringing in the ears (tinnitus)** | 2 (6.5%) | 11 (7.2%) | 3 (3.8%) | 6 (21.4%) | 0 (0.0%) | **0.0327** | NE |
| **Blurry vision** | 2 (6.5%) | 5 (3.3%) | 10 (12.5%) | 4 (14.3%) | 2 (3.8%) | 0.0844 | 0.5722 |
| **Dizziness** | 7 (22.6%) | 19 (12.5%) | 8 (10.0%) | 2 (7.1%) | 0 (0.0%) | **0.0327** | NE |
| **Cutaneous or vascular changes** | 1 (3.2%) | 1 (0.7%) | 0 (0.0%) | 0 (0.0%) | 0 (0.0%) | 0.5108 | NE |

| **Symptom** | **A n (%)** | **B n (%)** | **C n (%)** | **D n (%)** | **E n (%)** | **Fisher FDR** | **GEE FDR** |
| --- | --- | --- | --- | --- | --- | --- | --- |
| Nausea | 15 (48.4%) | 79 (52.0%) | 30 (37.5%) | 23 (82.1%) | 20 (37.7%) | **0.0008** | **0.0162** |
| Vomiting | 3 (9.7%) | 3 (2.0%) | 3 (3.8%) | 3 (10.7%) | 2 (3.8%) | 0.0779 | 0.2793 |
| Photophobia (concomitant) | 27 (87.1%) | 100 (65.8%) | 58 (72.5%) | 19 (67.9%) | 48 (90.6%) | **0.0025** | **0.0162** |
| Phonophobia (concomitant) | 19 (61.3%) | 70 (46.1%) | 45 (56.2%) | 19 (67.9%) | 36 (67.9%) | **0.0336** | 0.7449 |
| Osmophobia (concomitant) | 15 (48.4%) | 23 (15.1%) | 10 (12.5%) | 14 (50.0%) | 3 (5.7%) | **0.0001** | **0.0162** |
| Other concomitant | 10 (32.3%) | 19 (12.5%) | 21 (26.2%) | 3 (10.7%) | 2 (3.8%) | **0.0008** | **0.0045** |

***Supplementary Table S7. Distribution of concomitant symptoms across patient cohorts.*** *Values are expressed as n (%) of attacks within each cohort. Global differences across cohorts were assessed using Fisher’s exact test and logistic generalized estimating equations (GEE) accounting for within-patient clustering. P-values were adjusted for multiple comparisons using the Benjamini-Hochberg false discovery rate (FDR) procedure. Significant FDR-adjusted p-values are shown in bold.*

| **Symptom** | **A n (%)** | **B n (%)** | **C n (%)** | **D n (%)** | **E n (%)** | **Fisher FDR** | **GEE FDR** |
| --- | --- | --- | --- | --- | --- | --- | --- |
| Humor | 10 (32.3%) | 59 (38.8%) | 37 (46.2%) | 16 (57.1%) | 29 (54.7%) | 0.093 | 0.791 |
| Appetite | 12 (38.7%) | 52 (34.2%) | 14 (17.5%) | 17 (60.7%) | 17 (32.1%) | **0.002** | 0.333 |
| Craving | 7 (22.6%) | 21 (13.8%) | 7 (8.8%) | 9 (32.1%) | 5 (9.4%) | **0.033** | 0.333 |
| Cognitive | 5 (16.1%) | 55 (36.2%) | 18 (22.5%) | 17 (60.7%) | 17 (32.1%) | **0.002** | 0.513 |
| Sleep | 14 (45.2%) | 57 (37.5%) | 33 (41.2%) | 14 (50.0%) | 12 (22.6%) | 0.093 | 0.351 |
| Temperature | 11 (35.5%) | 48 (31.6%) | 9 (11.2%) | 15 (53.6%) | 10 (18.9%) | **0.000** | 0.333 |
| Yawning | 13 (41.9%) | 65 (42.8%) | 15 (18.8%) | 7 (25.0%) | 12 (22.6%) | **0.002** | 0.488 |

***Supplementary Table S8.*** *Distribution of postdromic symptoms across patient cohorts****.*** *Values are expressed as n (%) of attacks. Group A: high-intensity; Group B: mixed pattern; Group C: acute-onset; Group D: prolonged-intense; Group E: low-intensity. GEE analyses accounted for within-patient clustering, and p-values were FDR-adjusted.*

| **Patient profile** | **First medication relative to pain peak, mean ± SD (h)** | **n** |
| --- | --- | --- |
| A- High-intensity | 6.6 ± 23.2 | 29 |
| B- Mixed | 2.2 ± 18.2 | 129 |
| C- Acute-onset | 0.3 ± 2.3 | 63 |
| D- Prolonged-intense | **19.9 ± 28.3** | 24 |
| E- Low intensity | 3.1 ± 13.1 | 25 |

**Supplementary Table S9.** Timing of the first acute treatment relative to the pain peak according to patient profile. Values are expressed as mean ± SD hours relative to the pain peak; positive values indicate treatment after the peak. n represents the number of attacks with available timing data. Group A: high-intensity; Group B: mixed pattern; Group C: acute-onset; Group D: prolonged-intense; Group E: low-intensity.
